# Does E-commerce participation increase the use intensity of organic fertilizers in fruit production?–Evidence from China

**DOI:** 10.1371/journal.pone.0273160

**Published:** 2022-08-30

**Authors:** Cuicui Wang, Hua Wang, Chunping Xia, Abdelrahman Ali

**Affiliations:** 1 School of Economics and Management, Yantai University, Shandong, China; 2 College of Economics and Management, Huazhong Agricultural University, Hubei, China; 3 Agricultural Economics Department, Agricultural College, Fayoum University, Fayoum, Egypt; Universidad Nacional Autonoma de Nicaragua Leon, NICARAGUA

## Abstract

Commerce has had positive impacts on the whole agri-food value chain at different stages, it was developed rapidly in rural China in the past few years. E-commerce participation can promote the use intensity of organic fertilizers (OF) and this could achieve many benefits for different stakeholders including ensuring food safety, positive environmental impacts and promoting the adoption of green production technologies. Therefore, this study has used primary data collected from 733 fruit farmers in rural China to explore the impact of e-commerce participation on fruit farmers’ use intensity of (OF). Unlike previous studies investigating the dichotomous decision of (OF) adoption, this study captures the use intensity of (OF) from both input quantity and cost aspects. We employed an endogenous switching regression (ESR) model to address selectivity bias caused by observed and unobserved factors. The results show that e-commerce participation significantly increases the use intensity of (OF) in input quantity and cost by 19.48% and 29.50%, respectively. Heterogeneous analysis further reveals that compared to fruit farmers with a low e-commerce participation level, fruit farmers with a high e-commerce participation level have higher (OF) use intensity. The findings also show that risk preference, human capital, cultivated area, cooperative membership and government restraint mechanisms positively and significantly affect the probability of fruit farmers’ participation in e-commerce and fruit farmers’ use intensity of (OF). The results emphasize that e-commerce promotion is an efficient way to encourage farmers to adopt (OF), which help improve product quality and promote sustainable agricultural development.

## Introduction

Organic fertilizer (OF) is an environmentally friendly alternative technology to chemical fertilizers due to its positive impacts on the soil, animal and human health. It has the advantages of long-lasting fertilizer efficiency and sufficient nutrients [1, 2] also it increases organic matter, enhances biodiversity, and improves soil fertility [3, 4]. Moreover, empirical evidence based on micro-household-level data suggests that (OF) helps to increase crop yields [5]. Studies have shown that (OF) help farmers, especially fruit farmers, improve their agricultural products’ output and quality, thereby providing them a prerequisite for entering high-cost markets [4]. With the increasing attention and popularity of organic fruits among consumers, using (OF) has become an important way to improve fruit quality and efficiency. Therefore, it is necessary to explore the determinants of fruit farmers’ adoption of (OF).

Earlier studies investigating the determinants of (OF) use focused mainly on individual characteristics and capital endowments, farmers’ cognition, and government policies [6–12]. Apart from the factors, the market drive is also essential in the (OF) adoption. market drive is also essential in organic fertilizer adoption. A study conducted by Ma et al. [13] shows that cooperative members have advantages in reducing market search costs and improving bargaining power; these advantages can promote the use of organic fertilizers by farmers. Market economic returns are the main factor that drives farmers to protect the quality of farmland [14, 15]. Reducing trade costs helps promote the adoption of green production technologies by farmers, and higher adoption increases farmers’ income when trade costs are low [16].

E-commerce has positive impacts on the whole agro-food value chain at the different stages. Using the E-commerce in the agricultural marketing could achieve many benefits for different stakeholders and for the national economy [17, 18]. Thus, the innovation in the sales model of agricultural products on the demand side and the economic benefits it brings will in turn drive the development of agricultural production in new directions (such as organic production) on the supply side [1]. E-commerce can reduce intermediate links in agricultural product marketing and the time to deliver the products from the farm to the consumer, which could achieve the short agricultural supply and value chains [17, 19]. As mentioned above, the reduction of transaction costs can promote farmers to implement green production. Moreover, e-commerce can help broaden the channels through which farmers obtain information, alleviate the information asymmetry between buyers and sellers, and form a shared and transparent production mode [6, 17]. The existing literature has confirmed the positive effects of e-commerce on the income diversity of rural households [20], farm income [18], rural household digital credit [21–23], and rural household welfare [24]. However, to the best of our knowledge, no previous studies except Li et al. [6] have researched the impact of e-commerce on the adoption of organic fertilizers by farmers. By estimating a propensity score matching (PSM) model, Li et al. [6] analyzed the effect of e-commerce on the adoption of green production and found that e-commerce significantly promotes the adoption of organic fertilizers by households. However, this study fails to consider the unobserved selectivity bias issue of e-commerce due to the limitation of the PSM method.

In 2019, the quantity of local fruit production in China estimated around 774 million tons and the import volume was 7.09 million tons (its value was 11.3 Billion US dollars). When the export volume was 3.61 million tons (its value was 5.9 Billion US dollars) [25]. In terms of the market share of organic agricultural products in China, the production area of organic crops was 2.328 million hectares in 2019, and the sales of plant products were 3.427 billion yuan represents 5% of the total sales value from the organic agricultural products which were 67.821 billion yuan in the same year [26]. This percentage of organic food products projected to increase in the future to follow the global food system transformation which aims to transform the agro-food system in different countries to be more sustainable and resilience with the climate change [27, 28]. This highlights the importance of current study to provide an empirical evidence for the policymaker to identify the actual role of stakeholders’ E-commerce participation on the transforming from the conventional agricultural system to organic and sustainable agro-food system.

Previous studies have investigated only the overall average effect of e-commerce participation on fruit farmers’ use of (OF) [6]. However, with different levels of e-commerce participation, fruit farmers may obtain different levels of benefits, thereby leading to different effects of e-commerce on the use of (OF). Although, the importance of investigation the impact of e-commerce on the farmers adoption of (OF) the previous literature hasn’t clear explain these impacts. So this study aims to estimate the effect of e-commerce participation on fruit farmers’ use intensity of (OF) in China. We try to make important contributions to the literature from the following three aspects (the novelty of this study). First, unlike previous studies on the binary use decision of (OF) by farmers [6, 13, 29, 30], we analyze the effect of e-commerce participation on the use intensity of (OF) (i.e., commercial organic fertilizer and farmyard manure) from both input quantity and cost aspects because most fruit farmers in the surveyed areas use (OF), and the use intensity of (OF) can better reflect the differences in the use of (OF) by farmers. Second, we employ an endogenous switching regression (ESR) model to correct the selection bias issue associated with e-commerce participation by considering both observed factors (e.g., gender, education, cultivation years, and fruit cultivated area) and unobserved factors (i.e., personal preferences and psychological motivations) [31]. Third, we also investigate the heterogeneity of the impact of e-commerce on fruit farmers’ use intensity of (OF) keeping in view the level of participation in e-commerce.

## Theoretical framework and hypotheses

Agricultural e-commerce (AEC) is a business activity carried out through electronic data transmission technology that eliminates the time and space barriers that are associated with the information transmission of traditional transaction systems [32]. AEC helps connect farmers living in remote rural areas with large markets, thereby promoting the development of the rural economy. Moreover, AEC is crucial in increasing farmers’ income and reducing blindness in production [18]. These are the main ways that fruit farmers participate in AEC; First, fruit farmers participate in traditional e-commerce platforms (by using the local e-business shops) to sell agricultural products. For example, fruit farmers sell agricultural products through third-party e-commerce platforms, such as (*Taobao™ and JD*.*com™* etc.) [33, 34]. Second, fruit farmers rely on social media to participate in AEC, to find information about agricultural products and to conduct online trade. For example, farmers sell agricultural products through *WeChat*, live broadcasts, and community group buying. Moreover, AEC enables farmers to learn about the latest agricultural production technology and social development trends to improve farmers’ scientific and cultural qualities, and to reduce agricultural production risks [12, 35].

**Fig 1:**
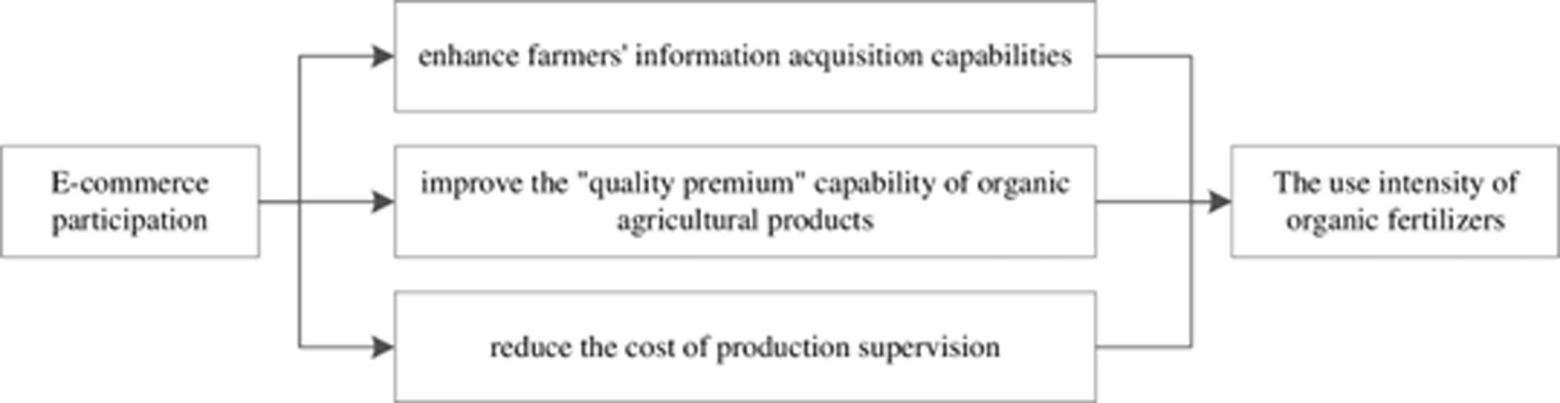
Pathways of E-commerce participation impacts on fruit farmers’ use intensity of organic fertilizers.

In this study, we expect that fruit farmers’ use intensity of (OF) is influenced by e-commerce participation in three pathways (see Fig 1). **First**, e-commerce participation can enhance farmers’ information acquisition capabilities by expanding information acquisition channels. With the increase in farmers’ understanding of (OF), the farmers can fully understand the positive role of (OF) in improving product quality, improving soil organic matter, and alleviating nonpoint source pollution, in that way facilitating the use intensity of (OF). E-commerce enables farmers to obtain information from the internet and e-commerce platforms, and communicate with other online supply chain participants (e.g., consumers, other vendors, and sales agents), thereby helping farmers obtain information and resources cheaply and quickly [36]. Therefore, e-commerce participation can improve farmers’ understanding and mastery of organic fertilizer use information, and reduce the transaction cost of organic fertilizer use technology [37, 38]. Furthermore, E-commerce participation helps to strengthen the communication between e-commerce farmers and other online merchants about the purchase price and source of (OF), which may have an impact on production costs. Therefore, e-commerce plays a vital role in increasing farmers’ professional knowledge about (OF) [37], optimizing the allocation of agricultural production factors [39], and increasing the use intensity of (OF) by farmers.

**Second**, e-commerce participation can improve the “quality premium” capability of organic agricultural products. E-commerce participation reduces intermediate links in agricultural product sales, increases the efficiency of the circulation of agricultural products, and improves market prices [40]. In other words, e-commerce can reduce the sharing of quality premiums by middlemen [41]. Moreover, e-commerce can alleviate information asymmetry [42], enhance the transparency of market and product information, and expand the range of markets for fruit, thereby helping play the role of the price mechanism in market competition [6]. Therefore, e-commerce can expand the sales channels of agricultural products and enables a green agricultural product to be a “high-quality product with a high price” for [6, 37], thereby improving farmers’ awareness and adoption of (OF). In addition, as the level of e-commerce participation of farmers increases, the greater the effect of the quality premium and consequently, the greater the use probability and intensity of (OF) by fruit farmers.

**Third**, e-commerce participation can reduce the cost of production supervision. E-commerce participation is an entrepreneurial behavior [6]. To ensure subsequent entrepreneurial performance, farmers participating in e-commerce are more concerned about the word of mouth about their products and about their personal reputation [6, 37]. E-commerce helps build and improve the interactive communication and after-sales mechanism between producers and consumers of agricultural products. E-commerce fruit farmers can directly interact with consumers through e-commerce platforms, especially social e-commerce platforms (e.g., *WeChat* and live broadcast), to learn about consumers’ market demands. Moreover, e-commerce fruit farmers can optimize production inputs (e.g., increase organic fertilizer inputs) and improve production performance according to the feedback market demand [37]. Additionally, consumers can learn about the production process and product form of agricultural products through the e-commerce platform [43], thus increasing the intimacy and trust between the buyers and sellers of agricultural products and monitoring the quality and safety of agricultural products to a certain extent. Therefore, with this real-time interactive communication, fruit farmers can proactively adopt (OF) and increase the use intensity of (OF) to obtain a reputation premium, thereby suppressing farmers’ opportunistic behavior and reducing production supervision costs.

Based on the previous presentation, we expected the stakeholders’ e-commerce participation has a positive impact on fruit farmers’ use intensity of organic fertilizers, which could accelerate their transformation to adopt the organic agriculture system. At the same time, that could achieve a lower agro-food production environmental footprint, besides achieving sustainable rural development through enhancing the income of small farmer to produce healthy and safety food [44].

Hypothesis 1: E-commerce participation positively affects fruit farmers’ use intensity of organic fertilizers.

## Agricultural products e-commerce overview, data source and methodology

In the following section, we explained an overview of agricultural products e-commerce, the data collection including (data source and sampling strategy), and variables (dependent and control variable), and econometric model description.

### The status of agricultural products e-commerce in China

**Fig 2:**
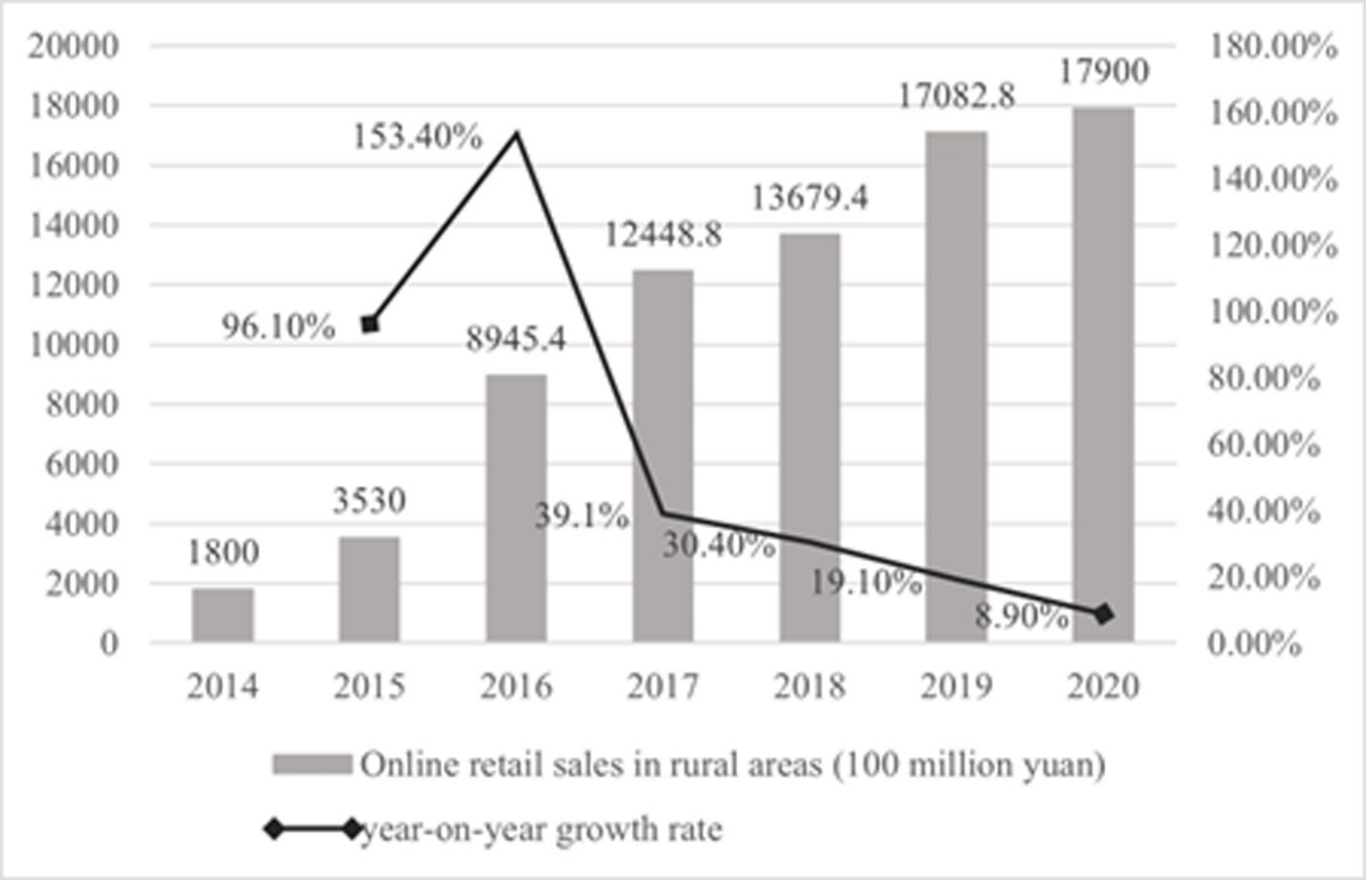
Rural network retail sales and year-on-year growth rate in China from 2014 to 2020. Source: Ministry of Commerce of the People’s Republic of China.

**Fig 3:**
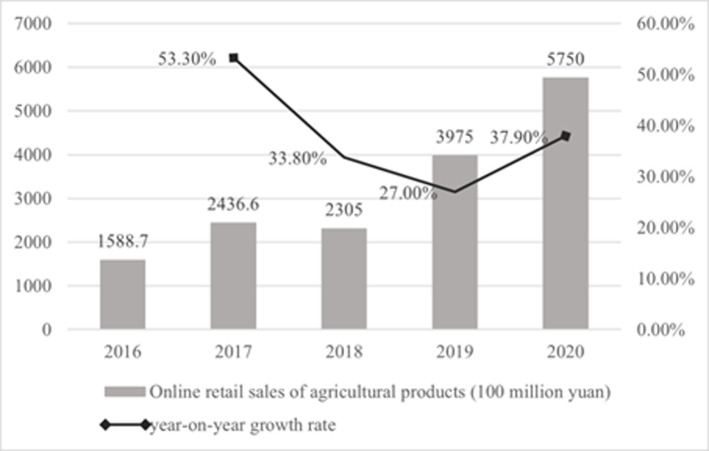
Agricultural products online retail sales and year-on-year growth rate in China from 2016 to 2020. Source: Ministry of Commerce of the People’s Republic of China.

With the rapid development of information and communication technology (ICT) and the improvement of logistics infrastructure, rural e-commerce and agricultural products e-commerce in China are developing rapidly [18]. According to the "2021 National County Digital Agriculture Rural E-Commerce Development Report", by the end of 2020, the number of netizens in rural China reached 309 million, accounting for 31.3% of the total netizens, an increase of about 54 million compared with March 2020, indicating that the scale of rural netizens continued to increase. According to data released by the Ministry of Commerce of the People’s Republic of China, the scale of rural e-commerce and the scale of agricultural products e-commerce are both continuously increasing (Figs 2 and 3). In 2020, rural online retail sales reached 1.79 trillion yuan, a year-on-year increase of 8.9%, accounting for 15.22% of the national online retail sales. Among them, the online retail sales of agricultural products reached 575 billion yuan, a year-on-year increase of 37.9%. Moreover, In 2020, The online retail sales of county-level agricultural products was 350.76 billion yuan, a year-on-year increase of 29.0%. The development of agricultural products e-commerce has entered a new stage of improving quality and growth.

With the improvement of living standards and the change and upgrading of consumption concepts, the per capita fruit consumption of Chinese residents has been increasing. According to the "2020–2021 Agricultural Products E-commerce Research Report", fruits ranked among the top ten in China’s agricultural products e-commerce sales category in 2019, which shows that consumers have an urgent and strong demand for fruits in agricultural product e-commerce. Moreover, new e-commerce sales models such as live streaming and community group buying continue to emerge during the COVID-19 pandemic, which further promotes the rapid development of fruit e-commerce. Therefore, this paper mainly takes fruit as the research object to analyze the influence of e-commerce participation on the use intensity of (OF).

### Data source and sampling strategy

The current study has used primary data that collected through a field survey of fruit farmers from November 2020 to April 2021. A multistage-stage sampling technique was used for data acquisition. The first stage is nonprobability sampling. In this stage, three provinces have been selected including (*Hubei*, *Shaanxi and Shandong*), four counties (*Zigui*, *Mei*, *Fufeng*, and *Pingyi*) were selected. The selection of survey counties was based chiefly on the following reasons. First, these areas are the dominant fruit production areas for fruit cultivation, and they represent a certain degree of the total fruit production in China. Specifically, *Zigui* County, *Hubei* province is rich in citrus and is known as the "Hometown of Navel Oranges in China". Wutai Yellow Peach in *Pingyi* County, Shandong province has a long history and is known as the "Hometown of Yellow Peach in China". The scale of kiwifruit cultivation in Shaanxi province ranks first in China, and *Meixian* and *Fufeng* Counties have a suitable climate and fertile soil, thus making these counties tone of the best kiwifruit-producing area. Furthermore, all three kinds of fruits are geographical indication products. Moreover, all four counties were successively selected as national e-commerce demonstration project regions, and have achieved certain levels of agricultural product e-commerce development with a certain number of fruit farmers sell fruit by participating in e-commerce. Second, the selected sample cities represent the level and mode of economic development in the central, western and eastern regions of China, respectively. In the second stage, using stratified random sampling, we randomly selected 1 to 3 towns from each city, then selected 2 to 5 villages from each town, and finally randomly selected 25 to 35 fruit farmers from each village.

Due to the lack of relevant information on the population of fruit farmers in the survey area in the survey area, we used the Cochran’s formula to define the number of samples [32]. Cochran’s formula is written as *n*_0_ = *pqZ*^2^/*e*^2^, where we assume a confidence level of 95% with a *Z*-value of 1.96, a probability of 0.5, and a margin of error *e* of 5%. Thus, we bring these values into the Cochran’s formula to define the minimum sample size, *n*_0_ = (0.5)(0.95)(1.96)^2^/(0.05)^2^ = 385. On this basis, this study relied on a sample size of 733 respondents to ensure precision.

Before the questionnaire survey, the interviewed farmers were asked whether their main source of income was the production and operation of the corresponding fruit industry, and whether they agreed to fill out the questionnaire anonymously for academic research. And a questionnaire survey was conducted after obtaining the oral consent of the fruit farmers. A structured questionnaire was used to collect relevant information on household and farm-level characteristics (e.g., age, gender, education, agricultural labor, fruit cultivated area, and agricultural income), e-commerce participation status, agricultural inputs status (e.g., organic fertilizers), fruit yield and sales price, and government policies (e.g., restraint mechanisms and government subsidies). Before the formal survey, we also modified the questionnaire based on the pre-investigation feedback. In order to better conduct face to face interviews, we also trained the enumerators. After checking and excluding the missing samples of the important key variables, we obtained 733 valid questionnaires, with an effective rate of 91.17%.

### Variables

The main goal of this study is to analyze the average and heterogeneous effects of e-commerce participation on fruit farmers’ use intensity of (OF).

#### Dependent variable

The outcome variables of this paper are the use intensity of (OF) in input quantity and cost. The use intensity (measured by quantity) of organic fertilizers (QOF) was captured through the “proportion of quantity input of (OF) to the total input of fertilizers in the previous year”, while the use intensity (measured by cost) of organic fertilizers (COF) was captured through the “proportion of cost input of (OF) to the total input of fertilizers in the previous year”. Here, organic fertilizer refers to commercial organic fertilizer, and farmyard manure (purchased and home-produced). Moreover, we convert farmyard manure produced by households according to the market price of the surveyed area and count the value into fruit farmers’ use of (OF) in input cost.

#### Key independent variable

The key independent variable in this paper is e-commerce participation. Following commonly used in the previous studies [6, 18, 19], a dichotomous variable is adopted to characterize e-commerce participation. The variable refers to “Do you participate in e-commerce to sell agricultural products?” (Participation methods: 1 third-party self-operated online store, 2 *WeChat* platforms, 3 live broadcast platforms, and 4 community group buying). In addition, to explore the heterogeneity of the impact of e-commerce participation on fruit farmers’ use intensity of (OF), we also consider the level of e-commerce participation, which refers to “what proportion of agricultural products sold (revenue generated) through e-commerce compared to the total agricultural products sales revenue?”

#### Control variables

Fruit farmers’ use intensity of (OF) is affected by both the farmers’ characteristics and external environmental factors [6, 9, 19, 32, 39, 45]. Therefore, we selected multiple control variables that consider respondents’ individual characteristics, such as gender, age, education, and risk preference. To consider the capital endowments perspective, we selected agricultural labor use, cultivation years, fruit cultivated area, agricultural income, political identity, and cooperative membership. To consider the external environment, we selected restraint mechanisms, government subsidies and brand construction as control variables. In addition, we also selected e-commerce training experience as the identifying instrumental variable (i.e., IV).

### Empirical strategy

Several studies have shown that farmers’ e-commerce participation behavior is endogenous [6, 19, 22] and that there are some unobservable factors, such as personal preferences and psychological motivations, that influence farmers’ participation in e-commerce and organic fertilizer adoption [6]. Therefore, this paper uses the ESR model to investigate the impact of e-commerce participation on fruit farmers’ use intensity of (OF).

The estimation of the ESR model involves the following two steps: First, the maximum likelihood method is used to estimate the decision-making equation and identify the factors influencing the participation behavior of fruit farmers in e-commerce (see Eq (1)). Second, the decision-making equation of the use intensity of (OF) for both participants and nonparticipants in e-commerce is established (see Eqs (2) and (3)):

$$P_{i}=\alpha Z_{i}+v_{i}$$ (1)

Where *P*_*i*_ represents the virtual variable of whether the fruit farmer participates in the e-commerce of agricultural products; *Z*_*i*_ denotes the various observable factors that affect the decision-making of the fruit farmer’s e-commerce; and *v*_*i*_ is the error term.

$$y_{1}=\eta_{1}X_{1}+\mu_{1}$$ (2)

$$y_{0}=\eta_{0}X_{0}+\mu_{0}$$ (3)

Where y_1_ and y_0_ represent the organic fertilizer use intensity of fruit farmers who are e-commerce participants and nonparticipants, respectively; X_i_ is a series of factors that affect the use intensity of (OF); and *μ*_1_ and *μ*_0_ are both error terms.

In the second stage, to eliminate the problem of selectivity bias due to unobservable factors, we substituted the inverse mills ratio and the covariance obtained in the first stage into two equations to obtain unbiased estimates of the parameters. Then, we estimated the average treatment effect (ATE) of the overall sample, e-commerce participants (treatment group) and nonparticipants (control group) (ATU). However, since both the overall sample and control groups contain samples that have been unaffected by e-commerce policies, the estimated results of ATE and ATU are of little importance. The most important thing is the average treatment effect of the treatment group (ATT) [46]. Therefore, we estimate only ATT:

$$ATT=E(y_{1}|P=1;\;X)-E(y_{0}|P=1;\;X)$$ (4)

In addition, Eq (1) should have at least one identifying instrumental variable that affects fruit farmers’ participation in e-commerce but does not directly affect farmers’ use intensity of (OF). The reason for selecting the identifying variable is that 44.94% of the fruit farmers participating in e-commerce participated in e-commerce training. Additionally, the content of e-commerce training focuses on e-commerce awareness and operation skills and thus does not directly affect fruit farmers’ use intensity of (OF).

## Results

Fruit farmers’ participation in e-commerce affects farmers’ use intensity of (OF) through different ways [11]. In the following section, the results of the analysis have been presented and discussed including the (descriptive analysis and the econometric model results).

### Descriptive analysis

Table 1 shows a descriptive analysis of the variables used in this study. The survey results show that the mean use intensity (QOF) is 0.376, while the mean use intensity (COF) is 0.447. Fruit farmers who participated in e-commerce accounted for 50.07% of the total sample. Participation levels (the proportion of agricultural product e-commerce sales revenue in the total agricultural product sales revenue) below 0.25 accounted for 41.69% of the total sample of fruit farmers participating in e-commerce. Statistics of individual characteristics of the sample respondents shown that the proportion of respondents who are men is approximately 56% and the proportion of respondents who are women is almost 46%. The average age of the sample respondents was 50 years, with a mean education level of 1.93 [47], mainly junior high school level and below. The cultivated fruit area owned by the sample farmers was 5.425 mu, with fruit-growing experience of almost 19 years. The proportion of agricultural income to the previous year of sample respondents was 60%. Approximately 92% of the sample respondents reported distinctive local area brands of agricultural products, only 19% farmers participated in farmer cooperatives, and 16% reported their family political membership. Approximately 19% of the sample farmers participated in e-commerce training.

**Table 1:** Definition and descriptive statistics.

Variable	Symbol indicators description	Mean	S.D.
**Dependent Variable**			
Use intensity (measured by quantity) of organic fertilizers	The proportion of quantity input of organic fertilizers to the total input of fertilizers in the previous year	0.376	0.144
Use intensity (measured by cost) of organic fertilizers	The proportion of cost input of organic fertilizers to the total input of fertilizers in the previous year	0.447	0.161
Fruit yield	Fruit yields (100 kg/ mu)^a^	25.623	5.673
**Key independent variable**			
E-commerce participation	Participation in e-commerce, 1 = yes; 0 = no	0.501	0.500
**Control variables**			
Gender	Gender, 1 = male; 0 = female	0.561	0.497
Age	Actual age (years old)	50.382	10.007
Education	1 = elementary school and below, 2 = junior high school, 3 = high school, 4 = associate degree, 5 = undergraduate and above	1.928	0.945
Risk preference	1 = risk aversion, 2 = risk neutral, 3 = risk preference	1.689	0.771
Agricultural labor	Number of family members engaged in the agricultural labor force (units)	2.181	0.809
Cultivation years	Number of years you have been cultivating fruit trees (years)	19.314	9.505
Fruit cultivated area	Planted area of fruit trees (mu)	5.425	4.084
Agricultural income	The ratio of agricultural income to the total household income in the previous year	0.610	0.277
Political identity	Whether your families have Party membership or cadre status? 1 = yes, 0 = no	0.157	0.364
Cooperative membership	Participation in farmer cooperative organization,. 1 = yes, 0 = no	0.188	0.391
Restraint mechanisms	Whether there is supervision, technical guidance or quality inspection (government/enterprise) in the process of agricultural production and sales, 1 = yes, 0 = no	0.109	0.321
Government subsidies	Subsidies for replacing chemical fertilizers with organic fertilizers, 1 = not at all, 2 = lesser degree, 3 = neutral, 4 = greater degree, 5 = absolutely	1.396	0.701
Brand construction	Does the local area have a distinctive brand of agricultural products? 1 = yes, 0 = no	0.918	0.274
**Identification variable**			
E-commerce training experience	Have you participated in e-commerce training in the past three years? 1 = yes, 0 = no	0.192	0.394

^a^1 mu = 1/15 hectare.

Moreover, to investigate the cross-statistical analysis, we divided the sample households into the following three categories on the basis of their participation in e-commerce: no participation, low level of participation and high level of participation in e-commerce. The results presented in Table 2 compare the use of (OF) on the basis of e-commerce participation. The results show the use intensity of (OF) in input quantity and cost (QOF and COF) both are increasing with increasing the level of e-commerce participation. Compared with respondents that did not participate in e-commerce, respondents with a high level of participation had a higher organic fertilizer use intensity. Measured by quantity, the use intensities of respondents with a high level of e-commerce participation and no level of e-commerce participation were 0.486 and 0.342, respectively; measured by cost, the use intensities were 0.579 and 0.396, respectively.

**Table 2:** Cross-statistical analysis of e-commerce participation and the use intensity of organic fertilizers.

	Number of households	The mean use intensity (measured by quantity) of organic fertilizers	The mean use intensity (measured by cost) of organic fertilizers
No participation	366	0.342	0.396
Low level (<0.434)	207	0.352	0.436
High level (≥0.434)	160	0.486	0.579

### Results of the ESR model

Tables 3 and 4 show the regression results of the ESR model on the influence of e-commerce participation on the use intensity of (OF) from both input quantity and cost aspects. The Wald test statistics of the joint independence of these equations show that the statistics are significantly different from zero, thus indicating that the three equations are dependent. In addition, *r*_1_ values in Tables 3 and 4 are both positively and statistically significant, thus showing that there are unobservable factors that affect e-commerce participation and the use intensity of (OF). Therefore, it is appropriate to use the ESR model in this paper to explore the effect of e-commerce participation on fruit farmers’ use intensity of OF.

**Table 3:** Determinants of e-commerce participation and determinants of the use intensity (measured by quantity) of OF.

Variable	Selection	The use intensity (measured by quantity) of organic fertilizers	
		Participants	Nonparticipants
Gender	0.043 (0.106)	0.014 (0.016)	0.021* (0.011)
Age	-0.015** (0.007)	-0.001 (0.001)	-0.001 (0.001)
Education	0.247*** (0.066)	0.040*** (0.009)	0.008 (0.009)
Risk preference	0.371*** (0.078)	0.083*** (0.012)	0.022** (0.011)
Agricultural labor	0.177*** (0.063)	0.026*** (0.009)	0.001 (0.009)
Cultivation years	0.017** (0.007)	0.002 (0.001)	0.001 (0.001)
Fruit cultivated area	-0.002 (0.015)	0.004** (0.002)	0.005*** (0.002)
Agricultural income	0.675*** (0.212)	0.089*** (0.032)	-0.000 (0.023)
Political identity	0.521*** (0.141)	0.034* (0.020)	0.020 (0.022)
Cooperative membership	0.123 (0.132)	0.039** (0.020)	0.035* (0.018)
Restraint mechanisms	0.202 (0.182)	0.056** (0.023)	0.087*** (0.028)
Government subsidies	0.016 (0.081)	0.012 (0.012)	0.010 (0.009)
Brand construction	0.923*** (0.272)	0.024 (0.055)	-0.003 (0.017)
E-commerce training experience	0.599*** (0.153)	—	—
Zigui	0.853*** (0.172)	0.160*** (0.030)	0.027 (0.021)
Fufeng/Mei	1.371*** (0.175)	0.128*** (0.031)	-0.051** (0.026)
Constant	-3.457*** (0.499)	-0.238*** (0.089)	0.252*** (0.051)
lns1	—	-1.771*** (0.046)	—
*r*_1_	—	0.950*** (0.019)	—
Lns0	—	—	-2.308*** (0.046)
*r*_0_	—	—	-0.219 (0.243)
Wald test *χ*^2^	47.72*** (*P-*value< 0.0001)		
Log- likelihood	220.662		

Note: ***, **, and * indicate significance at the 1%, 5%, and 10% levels, respectively. Standard error in parentheses.

**Table 4:** Determinants of e-commerce participation and determinants of the use intensity (measured by cost) of organic fertilizers.

Variable	Selection	The use intensity (measured by cost) of organic fertilizers	
		Participants	Nonparticipants
Gender	0.008 (0.109)	0.019 (0.017)	0.023* (0.013)
Age	-0.015** (0.007)	-0.002 (0.001)	-0.001 (0.001)
Education	0.235*** (0.069)	0.038*** (0.010)	0.004 (0.010)
Risk preference	0.356*** (0.081)	0.093*** (0.013)	0.020 (0.012)
Agricultural labor	0.190***(0.064)	0.024** (0.010)	-0.007 (0.010)
Cultivation years	0.017** (0.007)	0.002* (0.001)	0.001 (0.001)
Fruit cultivated area	-0.006 (0.015)	0.004* (0.002)	0.004** (0.002)
Agricultural income	0.773*** (0.218)	0.105*** (0.034)	-0.000 (0.026)
Political identity	0.561*** (0.147)	0.040* (0.022)	0.021 (0.025)
Cooperative membership	0.087 (0.138)	0.033 (0.021)	0.036* (0.021)
Restraint mechanisms	0.236 (0.195)	0.063*** (0.024)	0.072** (0.031)
Government subsidies	-0.006 (0.084)	0.014 (0.013)	0.010 (0.010)
Brand construction	0.903*** (0.271)	0.022 (0.064)	-0.014 (0.019)
E-commerce training experience	0.811*** (0.171)	—	—
Zigui	0.830*** (0.176)	0.183*** (0.034)	0.074*** (0.024)
Fufeng/Mei	1.388*** (0.178)	0.121*** (0.035)	-0.025 (0.030)
Constant	-3.436*** (0.512)	-0.172* (0.103)	0.306*** (0.058)
lns1	—	-1.732*** (0.053)	—
*r*_1_	—	0.897*** (0.040)	—
Lns0	—	—	-2.185*** (0.054)
*r*_0_	—	—	-0.304 (0.252)
Wald test *χ*^2^	21.74*** (P-value< 0.0001)		
Log- likelihood	142.485		

Note: ***, **, and * indicate significance at the 1%, 5%, and 10% levels, respectively. Standard error in parentheses.

#### Determinants of e-commerce participation among fruit farmers

The second columns of Tables 3 and 4 present the estimates of the determinants of fruit farmers’ e-commerce participation behavior estimated by Eq (1). The coefficients of the variables with the same name in the selection model in Tables 3 and 4 are explained together because these variables similarly affect the e-commerce participation behavior of fruit farmers. In both selection models, the age variable negatively affects e-commerce participation, showing that young and middle-aged respondents are more enthusiastic about learning new technologies and are willing to try new sales methods [43]. Education level statistically significantly and positively affects fruit farmers’ participation in e-commerce. The results accord with those of Kalambe [48], who found that farmers with higher levels of education are more likely to participate in e-commerce. The variable of risk preference is positively and statistically significant, thus suggesting that risk-taking farmers are more willing to participate in e-commerce. The agricultural labor variable, cultivation year’s variable and agricultural income variable all positively and statistically significantly affects fruit farmers’ participation in e-commerce. Moreover, the e-commerce training experience variable also positively and significantly affects fruit farmers’ participation in e-commerce. E-commerce training provides the opportunity for fruit farmers to learn and familiarize themselves with e-commerce operational skills, and creates a good e-commerce atmosphere, thereby making the farmers more likely to participate in e-commerce platforms. The variable representing brand building is positively and statistically significant. This estimation indicates that farmers are paying more attention to the role of brands in improving the recognition of their agricultural products and enhancing competitiveness [43]. The farmer location variables representing Fufeng and Zigui are also positively and statistically significant, thus showing that farmers in these cities are more willing to participate in e-commerce.

#### Determinants of fruit farmers’ use intensity of OF in input quantity and cost (QOF and COF)

Using Eqs (2) and (3), the estimates of the determinants of the use intensity (measured by quantity and cost) of organic fertilizer for e-commerce participants and nonparticipants are showed in the third and fourth columns of Tables 3 and 4. The results show (in Tables 3 and 4) the differences in the factors affecting the use intensity of OF for e-commerce participants and nonparticipants. The risk preference variable significantly positively affects the use intensity (measured by quantity and cost) of COF for e-commerce participants and the use intensity (measured by quantity) of QOF for nonparticipants. Farmers face many natural and social risks during production. Moreover, using OF entails technical risks. Therefore, the use intensity of OF for risk-loving fruit growers is higher [31]. Education positively and statistically significantly affects the use intensity (COF) for e-commerce participants. When farmers participate in e-commerce to obtain more economic benefits, farmers with better education levels have a higher intensity of use of OF [49]. However, the coefficient of age is statistically significant for fruit farmers who do not participate in e-commerce, thus suggesting that males are more inclined than females to use OF. This finding accords with the traditional background of the Chinese countryside.

The fruit cultivated area variable significantly positively affects the use intensity of OF for both e-commerce participants and nonparticipants from both input quantity and cost aspects, thereby indicating that the larger the planting area is, the more farmers are concerned about the quality improvement of agricultural products [23]. The Cooperative membership variable positively and statistically significantly affects the use intensity (QOF) for e-commerce participants and the use intensity (QOF and COF) for nonparticipants, thereby showing that farmers who are cooperative members are more likely to use OF. The possible reason is that agricultural cooperatives can provide information, resources and technical guidance for fruit growers [13, 50]. In addition, the agricultural labor, the agricultural income variable and the political identity variable all positively and statistically significantly affect the use intensity of OF for e-commerce participants from both input quantity and cost aspects. The cultivation years variable positively affects the use intensity (COF) for e-commerce participants.

The restraint mechanisms variable positively affects the use intensity (measured by quantity and cost) of OF for both e-commerce participants and nonparticipants. This finding indicates that supervision, technical guidance or quality inspection from the government or enterprises during agricultural production and sales can enhance the understanding of OF by fruit farmers and promote the use intensity of OF by fruit farmers [51]. The empirical results also confirm the impact of regional differences (e.g., economy, culture, and climate) on the use intensity of OF.

#### Results of treatment effects estimation

We further evaluated the average treatment effects on the treated (ATT), which show the impact of e-commerce participation on the use intensity of OF from both input quantity and cost aspects. The results (in Table 5) show that the use intensities (measured by quantity) of OF for e-commerce participants and nonparticipants are 0.41 and 0.344, respectively. The use intensities (measured by cost) of OF for e-commerce participants and nonparticipants are 0.496 and 0.383, respectively. The estimated ATT of e-commerce participation on the use intensities (measured by quantity and cost) of OF were 0.067 and 0.113, respectively. Moreover, the results show that concerning organic fertilizer use, the use intensities of fruit farmers participating in e-commerce significantly exceed the use intensities of fruit farmers who do not participate in e-commerce by 19.48% (in input quantity) and 29.50% (in input cost). Therefore, participating in e-commerce can increase fruit farmers’ use intensity of organic fertilizers, and Hypothesis 1 is verified.

**Table 5:** The average treatment effect of participating in e-commerce on fruit farmers’ use intensity of OF.

	Participants	Nonparticipants	ATT	Variety (%)
The use intensity (measured by quantity) of OF	0.411 (0.084)	0.344 (0.076)	0.067***	19.48
The use intensity (measured by cost) of OF	0.496 (0.099)	0.383 (0.077)	0.113***	29.50

Note: *** indicates the significance at the 1% levels. Standard error in parentheses.

#### Results of heterogeneous effects estimation

Economic benefits are crucial in promoting the transformation of the production and management methods of fruit farmers [19, 52]. The willingness of fruit farmers to change their production and management practices varies depending on the level of participation in e-commerce; consequently, fruit farmers’ use intensities of OF vary depending on the level of fruit farmers’ participation in e-commerce. We measured the level of fruit farmers’ participation in e-commerce by the proportion of e-commerce income from agricultural products to total agricultural product income. According to whether the level of participation was less than the average (0.434), we divided the fruit growers into a low group and a high group and included a sample of fruit growers who did not participate in e-commerce for regression analysis (in Table 6). We show that e-commerce increases the use intensity (measured by quantity) of OF by approximately 12 and 25% at low and high levels of participation, respectively. E-commerce participation also affects the use intensity (measured by cost) of OF monotonically, which increases the use intensity (measured by cost) of OF by 21.16% at a low level of participation and 30.41% at a high level of participation.

**Table 6:** Results of the heterogeneity analysis.

	Participation level	Participants	Nonparticipants	ATT	Variety (%)
The use intensity (measured by quantity QOF)	Low level	0.352(0.065)	0.313(0.066)	0.038***	12.14
	High level	0.485(0.094)	0.389(0.081)	0.096***	24.68
The use intensity (measured by cost COF)	Low level	0.434 (0.077)	0.344 (0.068)	0.090***	26.16
	High level	0.579 (0.100)	0.444 (0.080)	0.135***	30.41

Note: *** indicates the significance at the 1% levels. Standard error in parentheses.

### Additional analyses

As mentioned in the introduction, the use of (OF) can increase yields, which play a significant role in agricultural production [32]. Therefore, we further analyzed the effect of the use intensity of (OF) on fruit yield. Table 7 reports the OLS model regression results of the use intensity of (OF) on fruit yield. The results show that the use intensity (QOF and COF) statistically significantly and positively affect fruit yield, which indicated the use intensity of (OF) is expected to increase fruit yield and improve farm performance [5].

**Table 7:** The OLS model estimation results of the use intensity of (OF) on fruit yield.

Variable	Fruit yield	
The use intensity (measured by quantity) of organic fertilizers	0.995*** (0.059)	—
The use intensity (measured by cost) of organic fertilizers	—	0.907*** (0.052)
Control variables	controlled	controlled
*F*-value	32.98	40.65
R-squared	0.485	0.504

Note: *** indicates the significance at the 1% levels. Standard error in parentheses.

The dependent variables refer to the log-transformed forms of fruit yield.

## Discussion and conclusion

### Discussion

Unlike previous studies on agricultural green production that have considered the role of ICTs such as internet use and smartphones [39, 53, 54], we consider e-commerce, which provides more evidence for the impact of ICT technology, especially e-commerce, on the green production behavior of farmers. We conducted an empirical study on the relationship between e-commerce participation and the use intensity of (OF). We find: First, e-commerce participation positively affects the use intensity of (OF). E-commerce participation has expanded the sales channels of farmers, enhanced the transparency of market and product information, and improved farmers’ awareness of high-quality agricultural products and the sharing of the quality premium of green agricultural products, thereby prompting fruit farmers to adopt OF [6, 19]. Moreover, e-commerce participation has reduced the cost of external information and technical training for farmers and has changed farmers’ traditional production concepts, thus encouraging fruit farmers to use OF.

Second, e-commerce more strongly affects fruit farmers with a high level of e-commerce participation than those with a low level of e-commerce participation. In other words, the estimated ATT of e-commerce participation on fruit farmers’ use intensity of organic fertilizers increased with the level of participation in e-commerce. To a certain extent, the degree of e-commerce participation represents the sales ability of e-commerce. The stronger the sales ability and information ability of farmers are, then the higher the enthusiasm of farmers for constructing a quality traceability system and the stronger the motivation of farmers to use organic fertilizers [53]. Furthermore, the greater the degree of e-commerce participation is, the more economic benefits fruit farmers can achieve by realizing “high-quality with high-price” products, which can effectively stimulate fruit farmers to increase the use intensity of organic fertilizers. However, fruit farmers with a low level of participation in e-commerce have lower economic benefits, and the income effect of agricultural product e-commerce has a limited effect on improving their confidence in agricultural production and changing their production models.

### Conclusion and policy implications

In this paper, we used the farm level data to explore the effect of e-commerce participation on the use intensity (measured by quantity and cost) of OF by using the ESR model. Furthermore, we investigate the heterogeneous effects of e-commerce participation on fruit farmers’ use intensity of OF. The results show that e-commerce participation positively affects the use intensity of OF: e-commerce participation significantly increases organic fertilizer use intensity in input quantity and cost by 19.48% and 29.50%, respectively. E-commerce more strongly affects fruit farmers with a high level of e-commerce participation than those with a low level of e-commerce participation. Furthermore, e-commerce participation determinant results shows that the capital endowment and the external environment all affect e-commerce participation of fruit farmers, including age, education, risk preference, agriculture income, political identity and location dummy variables. In addition, fruit cultivated area, cooperative membership and government restraint mechanisms were found to significantly increase the use intensity of OF by fruit farmers. The additional analyses showed that the use intensity of (OF) significantly increases fruit yield.

The use of OF is important in improving product quality and promoting sustainable agricultural development. The theoretical analysis of this paper indicates that e-commerce participation can increase the use intensity of OF by enhancing fruit farmers’ information acquisition capabilities, improving the "quality premium" capability of organic agricultural products and reducing the cost of production supervision. Our results shows that the expansion of sales channels (i.e., e-commerce participation) can increase the use intensity of OF by fruit farmers. Therefore, the government should fully recognize the important role of e-commerce participation in increasing the use intensity of (OF) by fruit farmers. In practice, the government could reinforce the diffusion and participation of e-commerce, improve the e-commerce business ability of farmers, strengthen the construction of agricultural product brands, thereby promoting the synergistic improvement of e-commerce involvement and the use of (OF). Moreover, some online merchants may still be opportunistic, that is, use little or no organic fertilizer, and falsely report product quality information. Therefore, this paper proposes to strengthen the supervision of organic production and ensure the effectiveness of the realization of the green production quality premium. For example, the government can strengthen the construction and implementation of product quality rating and certification standards such as organic product certification and quality traceability systems. E-commerce platforms should raise and implement minimum requirements for the quality and safety of agricultural products, and pay close attention to consumer feedback. Furthermore, it is necessary to pay attention to the leading role of farmers’ professional cooperatives in the promotion of organic fertilizers due to its high efficiency of using production inputs and its collective action character [13].

Our study is based on cross-sectional data of 733 fruit farmers in China. E-commerce of agricultural products and the use of (OF) continue to proliferate over time. Due to data limitations, we have no way to capture the dynamic impact of e-commerce participation on the use intensity of (OF). However, we believe that it is a promising field. Therefore, panel data can be formed in the future for longitudinal comparison. Furthermore, we only analyzed the effect of the use intensity of (OF) on fruit yield in additional analyses. Due to data limitations, we are unable to capture the effect of the use intensity of (OF) on fruit production efficiency. Improving agricultural productivity and agricultural efficiency plays an important role in agricultural production [55–58]. OF is an input that can increase productivity, and its impact on productivity varies with other inputs such as labor. Therefore, analyzing the impact of the use intensity of (OF) on fruit production efficiency, or jointly modeling the effect of e-commerce participation and the use intensity of (OF) on fruit production efficiency is another interesting extension of future research.

## Supporting information

S1 File (DOCX)
