# Supplementary material for: Does E-commerce participation increase the use intensity of organic fertilizers in fruit production?–Evidence from China
Source: PLoS One. 2022 Aug 30;17(8):e0273160. doi: 10.1371/journal.pone.0273160 (PMC9426888; doi:10.1371/journal.pone.0273160)
Supplement: S1 File — (DOCX) [file pone.0273160.s001.docx]

**果农电商参与和有机肥使用调查问卷**

**A Questionnaire on E-commerce Participation and Organic Fertilizer Use of Fruit Farmers**

**(Parts related to the content of the study)**

尊敬的朋友：

您好！我们目前正在完成一项关于农产品电商和有机肥使用的课题研究，需要您配合我们完成以下相关问题，调查采取不记名形式，数据仅作为科学研究使用。您的信息和隐私是受保护的。感谢您的支持与配合！

选择题请在相应选项前打“√”，填空题请在相应横向上填写。

Dear Friends:

Hello! We are currently completing a research project on e-commerce of agricultural products and the use of organic fertilizers, and we need you to cooperate with us to complete the following related questions. The survey is anonymous, and the data is used for scientific research only. Your information and privacy are protected. Thank you.

For multiple-choice questions, please tick "√" before the corresponding option, and for fill-in-the-blank questions, please fill in the corresponding horizontal direction.

调查地点：____________省____________市（县）___________镇（乡）_____________村

Survey location (Village/ County/ City/ Province): __________________________________________________

1.个体和家庭特征/ Individual and family information

（1）性别（）

What is your sex? ()

1.男/ Male 2.女/ Female

（2）年龄_____岁。

How old are you? _____ years

（3）您是户主吗？（）

Are you a household head? ()

1.是/ Yes 2.否/ No

（4）您的受教育水平是？（）

What is your education level? ()

1.小学及以下/ elementary school and below 2.初中/ junior high school

3.高中（含中专、职高）/ high school 4.专科/ associate degree

5.本科及以上/ undergraduate and above

（5）若有1000元用来投资，会收到五种可能的回报，您更喜欢哪一种？（）

If you invest 1,000 yuan, you will receive five possible returns. Which one do you prefer? ()

1.稳定的1000元/ Stable 1000 yuan

2.一半的可能是900元和一半的可能是1600元/ Half may be 900 yuan and half may be 1600 yuan

3.一半的可能是800元和一半的可能是2000元/ Half may be 800 yuan and half may be 2000 yuan

4.一半的可能是400元和一半的可能是3000元/ Half may be 400 yuan and half may be 3000 yuan

5.一半的可能是0元和一半的可能是4000元/ Half may be 0 yuan and half may be 4000 yuan

（6）家中是否有村干部、党员等？（）

Whether your families have Party membership or cadre status()

1.是/ Yes 2.否/ No

（7）从事果树栽培_____年。

How many years you have been cultivating fruit trees ? _____years

（8）您家共有劳动人口数量 人（15岁-65岁），从事农业的人数_____人。

Numbers of labor in your family is _____(15-65 years old). Number of laborers engaged in agriculture _____.

1. 您家水果栽培面积 亩。

Planted area of fruit trees ( mu).

（10）去年您家总收入_____元，其中，农业收入（水果种植收入）_____元。

Annual household income of your family last year is _____ yuan. Agricultural income (fruit farming income) _____ yuan.

（11）您家是否参加了农业合作社?（）

Did your family participate in an agricultural cooperative? ()

1.是/ Yes 2.否/ No

（12）本村有农村电商服务站?（）

Whether there is a rural e-commerce service station in the village? ()

A.是/ Yes B.否/ No

（13）您家手机、电脑和宽带安装情况（）

The installation of mobile phones, computers and broadband in your home.()

1.无配备/ None 2.配备不足/ Not enough equipment. 3.一般/ Equipped in general 4.比较齐全/ Equipped relatively complete 5.非常齐全/ Very well equipped

2.农业生产、品牌建设和政策情况Planting, brand building and policy information

（1）去年肥料投入情况/ The input of fertilizers last year

| 总肥料投入数量/公斤  the total input quantity of fertilizers/ kg | 有机肥投入数量（包括农家肥）/公斤  quantity input of organic fertilizers (include farmyard manure)/ kg | 有机肥投入占比  Proportion |
| --- | --- | --- |
|  |  |  |
| 总肥料投入成本/元  the total input cost of fertilizers/ yuan | 有机肥投入成本（包括农家肥）/元  cost input of organic fertilizers(include farmyard manure)/ yuan | 有机肥投入占比  Proportion |

（2）您家是否参与农产品电商销售（参与方式：①包括当地电商平台在内的第三方自营网店；②微商、微信朋友圈及QQ空间；③直播电商平台；④社区团购）

Do you participate in e-commerce to sell agricultural products?" (Participation methods: 1 third-party self-operated online store, 2 WeChat platforms, 3 live broadcast platforms, and 4 community group buying).

1.是/ Yes 2.否/ No

| 水果  种类  Types of fruits | 面积  Area | 亩产量  Yield per mu | 电商销售  E-commerce sales | | | 传统渠道（收购商收购等）traditional channels |
| --- | --- | --- | --- | --- | --- | --- |
|  |  |  | 售价  selling price | 销量  Sales quantity | 销售额Amount of sales | 售价  selling price |
|  |  |  |  |  |  |  |
|  |  |  |  |  |  |  |
|  |  |  |  |  |  |  |

（3）农业生产和销售过程中是否有监管/指导或质量检测（政府或企业）？

Whether there is supervision, technical guidance or quality inspection (government/enterprise) in the process of agricultural production and sales

1.是/ Yes 2.否/ No

（4）有机肥替代化肥的补贴力度：

Subsidies for replacing chemical fertilizers with organic fertilizers:

1. 没有/ not at all 2. 不大/ lesser degree 3. 一般/ neutral 4. 较大/ greater degree 5. 力度很大/ absolutely

（5）当地是否具有特色农产品品牌？

Does the local area have a distinctive brand of agricultural products?

1.是/ Yes 2.否/ No

（6）近三年是否参加过电商培训？

Have you participated in e-commerce training in the past three years?

1.是/ Yes 2.否/ No
